# Marked hemoglobin mass expansion and plasma volume contraction in Sherpas acclimatizing to 5,400 m altitude

**DOI:** 10.1152/japplphysiol.00247.2024

**Published:** 2024-11-07

**Authors:** Johanna Roche, Santosh Baniya, Suraj Bhatta, Sachin Subedi, Hannes Gatterer, Peter Rasmussen, Matthias Peter Hilty, Anne-Aylin Sigg, Santosh Timalsina, Christoph Siebenmann

**Affiliations:** ^1^Institute of Mountain Emergency Medicine, Eurac Research, Bolzano, Italy; ^2^Pokhara Academy of Health Sciences, Pokhara, Nepal; ^3^Mountain Medicine Society of Nepal, Kathmandu, Nepal; ^4^Genmab A/S, Valby, Denmark; ^5^Institute of Intensive Care Medicine, University Hospital Zurich, Switzerland; ^6^Department of Biochemistry, Chitwan Medical College (CMC), Bharatpur, Nepal

**Keywords:** hematocrit, hemoglobin, hypoxia, plasma volume, sherpas

## Abstract

In lowlanders, high altitude (HA) acclimatization induces hemoconcentration by reducing plasma volume (PV) and increasing total hemoglobin mass (Hb_mass_). Conversely, Tibetan highlanders living at HA are reported to have a similar hemoglobin concentration ([Hb]) as lowlanders near sea level, and we investigated whether this reflects alterations in the PV or the Hb_mass_ response to HA. Baseline assessment of PV and Hb_mass_ was performed by carbon monoxide rebreathing at low altitudes (∼1,400 m) in Sherpas (an ethnic group of Tibetans living in Nepal) and native lowlanders. Participants then ascended to the Everest Base Camp (EBC) (5,400 m), where further measurements were performed after ∼2 days (EBC 1) and ∼6 wk (EBC 2). While on EBC 1 an increase in [Hb] was observed in lowlanders (*P* = 0.004) but not in Sherpas (*P* = 0.179), marked increases in [Hb] were observed in both groups on EBC 2 (*P* < 0.001). On EBC 1, Hb_mass_ (Sherpas, *P* = 0.393; lowlanders, *P* = 0.123) and PV (Sherpas, *P* = 0.348; lowlanders, *P* = 0.172) were not different from baseline in either group, while circulating erythropoietin was increased in both groups (*P* < 0.001). On EBC 2, large increases in Hb_mass_ and reductions in PV were observed along with elevated circulating erythropoietin in both groups (all *P* < 0.002). Neither the increases in erythropoietin on EBC 1 (*P* = 0.846) or EBC 2 (*P* = 0.564) nor the expansion of Hb_mass_ (*P* = 0.771) or reduction in PV (*P* = 0.099) on EBC 2 differed between the groups. We conclude that the hematological response of Sherpas to extended exposure to very high altitudes does not fundamentally differ from that of native lowlanders.

**NEW & NOTEWORTHY** We measured the hematological response to ∼6 wk exposure to an altitude of 5,400 m in Sherpa highland natives and Nepalese lowlanders. While the increase in hemoglobin concentration at high altitudes tended to be smaller in Sherpas than in lowlanders, the two groups experienced a similar reduction in plasma volume and increase in hemoglobin mass. We conclude that the hematological response of Sherpas to high-altitude exposure does not fundamentally differ from that of lowlanders.

## Graphical abstract

**Figure:**
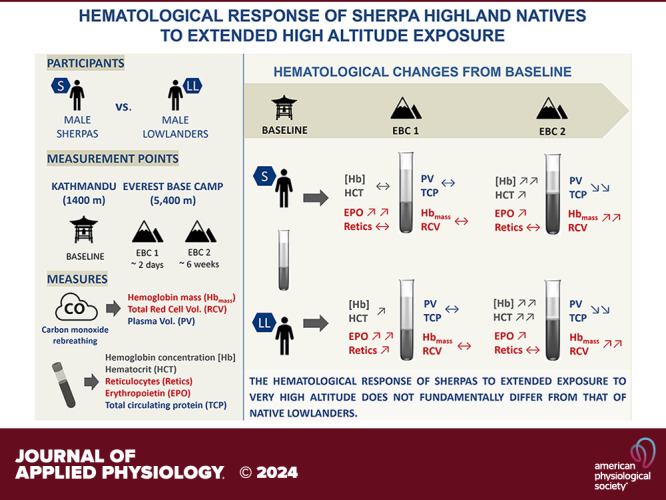

## INTRODUCTION

Lowlanders acclimatizing to the hypoxia of high altitude (HA) experience hemoconcentration resulting from a rapid reduction in plasma volume (PV) accompanied by a delayed expansion of total hemoglobin mass (Hb_mass_) (1, 2). Conversely, Tibetan highlanders, who have lived at HA for many generations (3), are reported to have a hemoglobin concentration ([Hb]) that is similar to or only slightly higher than that of lowland populations living near sea level (3–5). The absence of more pronounced hemoconcentration in Tibetans is widely attributed to genetic modifications blunting hypoxia-induced erythropoietin release and thus Hb_mass_ expansion at HA (6, 7). This explanation was extended by a recent study comparing Hb_mass_ and intravascular volumes between Sherpas (an ethnic group of Tibetan highlanders that has emigrated to Nepal), Andean highlanders, and lowlanders of European descent (5). While Hb_mass_ in the Sherpas was only mildly higher than in the lowlanders, and markedly lower than in the Andeans, the PV of the Sherpas exceeded that of the Andeans by ∼25% and even tended to be larger than that of the lowlanders. These findings suggest that the Tibetan response to HA hypoxia is characterized not only by a blunted increase in erythropoiesis but also by a mitigated reduction or even an increase in PV. Nevertheless, conclusions are precluded by the cross-sectional design of the study that did not include baseline measurements at low altitudes. A further limitation was that the altitude at which testing was performed and the duration of exposure to that altitude before testing differed between the Sherpas and the Andeans. Finally, it remains unclear whether different lifestyles (e.g., physical activity, diet, water intake) contributed to the differences between the studied populations.

The aim of the present study was to overcome these limitations by investigating the hematological response to HA in Sherpas with a longitudinal protocol and comparing it to that of native lowlanders with a similar lifestyle and undergoing the same HA exposure. Baseline measurements were conducted at the relatively low altitude of Kathmandu (Nepal, ∼1,400 m). Participants then ascended to the Everest Base Camp (EBC, ∼5,400 m), where further measurements were performed during the first week (EBC 1) and after ∼6 wk (EBC 2). Based on the paradigm that genetic modifications blunt the erythropoietic response to hypoxia in Sherpas, we hypothesized that *1*) on EBC 1, the increase in circulating erythropoietin from baseline values would be smaller in Sherpas than in lowlanders; and *2*) on EBC 2, the increase in Hb_mass_ from baseline values would be smaller in Sherpas than in lowlanders. Based on the recent study where the PV of Sherpas at HA was not smaller than that of lowlanders at sea level (5), we further hypothesized that *3*) the PV of Sherpas would not be reduced from baseline values on either time point in the EBC.

## MATERIALS AND METHODS

This study was approved by the Nepal Health Research Council (NHRC, Ref. 531/2022 P) and conducted in accordance with the Declaration of Helsinki (except for registration in a database).

### Participants

Individuals living in Kathmandu, or nearby villages at a similar altitude, and scheduled to work as EBC support staff during the upcoming climbing season, were recruited by contacting local trekking/climbing organizers. Initially, 35 males (18 Sherpas and 17 lowlanders) gave written informed consent to study participation after receiving written and oral information (in Nepali) from a local investigator and after being offered the opportunity to ask questions. Before baseline testing, all participants had lived at the altitude of Kathmandu for >2 mo except for one Sherpa (who dropped out after the baseline measurements) and one lowlander, both of whom had only resided at that altitude for 3 wk after spending the preceding weeks at ∼3,000 m. These participants were included since the effects of exposure to ∼3,000 m altitude on Hb_mass_ and intravascular volumes are expected to vanish within 2 wk after return to low altitude (8).

**Figure 1.**
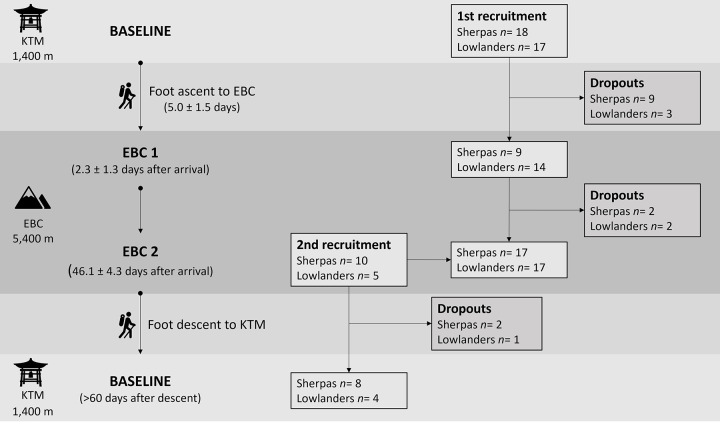
Protocol overview with number of participants and dropouts at each measurement time point. First recruitment was performed before the initial baseline testing in Kathmandu (KTM). Due to a large number of dropouts, a second round of recruitment was performed before the second measurement time point in the Everest Base Camp (EBC 2). The newly recruited participants had spent a similar number of days in the EBC as the originally recruited participants. Baseline measurements were performed for the newly recruited participants after the end of their sojourn in the EBC. Changes from baseline could thus be assessed on the first time point in the EBC (EBC 1) in 9 Sherpas and 14 lowlanders, and on EBC 2 in 15 Sherpas and 16 lowlanders, respectively.

On EBC 1, only 23 of the originally enrolled participants (9 Sherpas, 14 lowlanders) could be retested as the remaining 12 either did not reach the EBC due to illness or change of work assignment or could not be recontacted. On EBC 2, another four of the originally enrolled participants could not be retested for the same reasons, leaving only 7 Sherpas and 12 lowlanders. Accordingly, we recruited 15 additional participants (10 Sherpas, 5 lowlanders) who had spent a similar time in the EBC as the originally enrolled participants and declared to descend to the altitude of Kathmandu after the climbing season. Twelve of these additional participants (8 Sherpas, 4 lowlanders) later reported to “post-intervention” baseline measurements that were performed in Kathmandu >2 mo after the termination of the climbing season. The number of participants tested at each time point along with the number of dropouts is illustrated together with the study protocol (see next paragraph) in Fig. 1 and the participants’ anthropometrics in Table 1.

**Table 1.** Participant characteristics

	*Kathmandu*	*EBC 1*	*EBC 2*
Number of participants			
Sherpas	26	9	17
Lowlanders	21	14	17
Number of smokers			
Sherpas	7	0	6
Lowlanders	6	3	7
Baseline body mass, kg			
Sherpas	67.5 ± 11.0	67.1 ± 10.8	67.0 ± 8.7
Lowlanders	68.8 ± 8.2	69.7 ± 8.6	67.9 ± 8.4
Baseline height, m			
Sherpas	1.64 ± 0.05	1.66 ± 0.05	1.65 ± 0.06
Lowlanders	1.66 ± 0.05	1.67 ± 0.05	1.65 ± 0.05
Baseline age, yr			
Sherpas	35.3 ± 14.6	39.6 ± 13.6	34.6 ± 13.0
Lowlanders	33.1 ± 11.3	34.1 ± 11.5	33.5 ± 12.1
Days after arrival in the EBC, days			
Sherpas	N/A	3.0 ± 1.8	46.6 ± 5.0
Lowlanders	N/A	1.9 ± 0.6	46.2 ± 4.1

The number of participants tested during the baseline measurements in Kathmandu and on the two measurement time points in the EBC is provided along with their baseline characteristics. “Days after arrival in the EBC” specifies the number of days between the arrival in the EBC (reached by foot ascent over the course of 5.0 ± 1.5 days) and the EBC 1 and EBC 2 measurements, respectively. ERC, Everest Base Camp.

Participants were healthy and free of medication, except for one Sherpa who had diabetes and adhered to metformin treatment. Participants of the Sherpa group self-identified as Sherpas, and their identity cards confirmed that they, as well as their fathers and grandfathers, all carried the last name “Sherpa” (in Nepal, surnames are often used to indicate a person’s caste or ethnicity). The lowlander group included Nepalese from the Brahmin, Chhetri, Tamang, Gurung, and Rai ethnic groups, as well as one Western European member of the research team.

### Protocol

Baseline measurements in Kathmandu started with the assessment of peripheral oxygen saturation and heart rate by a pulse oximeter (Nonin 3150 Wrist Ox2, Plymouth, MN), the evaluation of symptoms of acute mountain sickness by the Lake Louise questionnaire (9), and the collection of venous blood for analysis of circulating erythropoietin, reticulocyte, and protein concentrations. Subsequently, Hb_mass_ and intravascular volumes were determined by carbon monoxide (CO) rebreathing.

The measurements in the EBC included the same procedures as the baseline measurements. Participants reached the EBC over the course of a multiday trek (Sherpas, 4.9 ± 1.4 days; lowlanders, 5.1 ± 1.6 days) starting at altitudes between 2,500 and 3,000 m. The EBC 1 measurements were performed during the first week, and the EBC 2 measurements 39–55 days after the participants’ arrival in the EBC, respectively. The time between the arrival and the measurements in the EBC is presented for both groups in Table 1. Most participants spent the entire time between arrival in the EBC and the EBC 2 measurement between 5,000 and 5,400 m; however, there were some exceptions: for work purposes, four participants (3 Sherpas, 1 lowlander) descended by foot to Lukla (2,860 m), where they spent 1–4 nights, and then returned to the EBC over the course of 2–8 days. Another three participants (1 Sherpa, 2 lowlanders) descended to and spent 1–3 nights at altitudes between 4,000 and 4,600 m before reascending to the EBC.

### Carbon Monoxide Rebreathing

We used a slightly modified version of our previously described CO rebreathing protocol (10): after consuming 0.5 L of water, participants rested supine for 20 min, whereafter 1 mL of venous blood was collected. Participants were then fitted with a mouthpiece through which they breathed pure oxygen from a Douglas bag for 1 min to washout nitrogen from the airways. Although in our usual protocol, this step lasts 4 min (10), the duration was shortened to reduce the expenditure of oxygen. Participants were subsequently switched via a sliding valve to an oxygen-filled rebreathing circuit containing a 6 L counterlung and a carbon dioxide scrubber. In Kathmandu, a CO volume corresponding to ∼1.5 mL per kg bodyweight was then injected into the circuit and rebreathed for 10 min. In the EBC, the injected CO volume was increased by ∼50% to compensate for the reduced barometric pressure. Another milliliter of venous blood was collected at the end of the CO rebreathing. Both blood samples were analyzed in quadruplicate in a hemoximeter (ABL90 FLEX, Radiometer, Copenhagen, Denmark) for carboxyhemoglobin fraction (%HbCO) and [Hb], whereas hematocrit was determined at least in duplicate by the micromethod. Hb_mass_ was derived from the administered CO dose and the resulting increase in %HbCO, and intravascular volumes were computed by integration of [Hb] and hematocrit (10). Note that we usually collect the first blood sample immediately before CO administration. In the current study, we collected it before the onset of oxygen breathing to further reduce oxygen expenditure and to prevent a potential influence of inhaled oxygen on circulating erythropoietin, which was measured in blood collected at the same time point (see next paragraph). Since the half-life of CO in venous blood during oxygen breathing exceeds 1 h (11), we do not expect that the %HbCO measured in the first blood sample was affected by the subsequent 1 min bout of oxygen breathing.

### Erythropoietin, Reticulocyte, and Plasma Protein Concentration

Three milliliters of venous blood were collected in a serum-separating tube. The blood was allowed to clot for 20–30 min, centrifuged at 4,000 rpm for 8–10 min, and then stored on ice. Another 3 mL was collected in an EDTA-coated tube and also stored on ice. All these samples were collected during the CO rebreathing procedure at the end of the 20-min resting period (i.e., before administration of oxygen). Stored samples were transported to an ISO 15189 accredited clinical laboratory in Kathmandu within 48 h after collection (transport from the EBC occurred by helicopter), where the following analyses were performed: serum total protein and erythropoietin concentrations were estimated by end-point colorimetric assay (Biuret method) and chemiluminescence immunoassay, respectively. Reticulocyte count was assessed using the manual method (12).

### Statistics

To investigate the main differences between results collected in Kathmandu and the EBC, a mixed model repeated measures (MMRM) analysis was conducted (note that values for circulating erythropoietin concentration were log-transformed before analysis as visual inspection indicated non-normal distribution and a large difference in variance between the groups). The MMRM analysis included ethnicity and was adjusted for order effects. Due to the large number of dropouts and resulting variation in number of participants tested at the different time points, we present absolute values for all participants tested at a given time point for descriptive purposes only and without statistical testing, whereas comparisons of the effects of HA between Sherpas and lowlanders are inferred by changes from baseline (Kathmandu) on EBC 1 and EBC 2, respectively. These were evaluated by post-hoc pairwise comparisons with *P*-values corrected by Tukey’s method for multiple comparisons. Since MMRM analysis handles missing data well (13), and answering our individual hypotheses required comparing changes from baseline between Sherpas and lowlanders either on EBC 1 or EBC 2, we included all participants in the analysis for whom we have measured at least one of these changes regardless of whether the other change was measured as well. All statistical analyses were conducted using SAS 9.4 (Cary, NC). Unless individual values are presented, results are reported as means ± SD. Statistical significance was set at *P* < 0.05 for all tests. Effect sizes were calculated for HA-induced changes in the main outcome variables ([Hb], Hb_mass_, and PV) as Cohen’s *d*.

## RESULTS

All participants who were tested in the EBC tolerated the HA well as indicated by their Lake Louise scores, which were all ≤1. Absolute values measured in Kathmandu and on the two EBC time points in all participants enrolled at a given time point are presented in Table 2.

**Table 2.** Absolute values measured in Kathmandu and the EBC

	*Kathmandu*	*EBC 1*	*EBC 2*
Number of participants			
Sherpas	26	9	17
Lowlanders	21	14	17
$\text{S}{\text{p}}_{{\text{O}}_{2}}$, %			
Sherpas	95.5 ± 1.6	78.6 ± 4.2	79.6 ± 3.2
Lowlanders	95.3 ± 1.3	79.2 ± 4.0	79.8 ± 4.0
Heart rate, beats min^−1^			
Sherpas	74.3 ± 10.7	93.1 ± 14.0	84.9 ± 11.5
Lowlanders	76.5 ± 14.3	89.6 ± 17.5	94.6 ± 15.7
[Hb], g L^−1^			
Sherpas	146 ± 12	147 ± 9	182 ± 11
Lowlanders	146 ± 11	152 ± 12	190 ± 14
Hematocrit, %			
Sherpas	43.5 ± 4.2	44.3 ± 3.7	55.7 ± 3.8
Lowlanders	43.8 ± 3.4	45.6 ± 3.3	58.7 ± 4.4
Hb_mass_, g			
Sherpas	744 ± 102	758 ± 77	972 ± 204
Lowlanders	754 ± 136	804 ± 161	993 ± 176
Hb_mass_ relative to body mass, g kg^−1^			
Sherpas	11.2 ± 1.7	11.5 ± 1.4	14.5 ± 1.9
Lowlanders	11.0 ± 1.7	11.5 ± 1.9	14.7 ± 2.4
PV, L			
Sherpas	2.89 ± 0.44	2.91 ± 0.49	2.35 ± 0.43
Lowlanders	2.90 ± 0.46	2.87 ± 0.49	2.15 ± 0.36
PV relative to body mass, L kg^−1^			
Sherpas	43.3 ± 6.2	43.6 ± 5.6	35.1 ± 4.2
Lowlanders	42.5 ± 6.5	41.4 ± 5.9	31.9 ± 5.1
RCV, L			
Sherpas	2.22 ± 0.31	2.29 ± 0.20	2.98 ± 0.66
Lowlanders	2.27 ± 0.41	2.41 ± 0.45	3.07 ± 0.51
RCV relative to body mass, L kg^−1^			
Sherpas	33.5 ± 5.5	34.7 ± 4.8	44.7 ± 6.2
Lowlanders	33.1 ± 5.1	34.7 ± 5.4	45.5 ± 7.2
BV, L			
Sherpas	5.11 ± 0.60	5.20 ± 0.63	5.32 ± 1.00
Lowlanders	5.17 ± 0.81	5.28 ± 0.88	5.22 ± 0.74
BV relative to body mass, g kg^−1^			
Sherpas	76.8 ± 9.9	78.3 ± 8.8	79.8 ± 8.1
Lowlanders	75.6 ± 10.4	76.0 ± 10.3	77.5 ± 10.5
Erythropoietin concentration, IU L^−1^			
Sherpas	8.42 ± 3.92	138 ± 193	28.0 ± 47.9
Lowlanders	9.58 ± 3.16	126 ± 215	26.5 ± 26.5
Reticulocyte concentration, %			
Sherpas	1.75 ± 0.75	3.37 ± 2.43	2.18 ± 1.11
Lowlanders	1.38 ± 0.55	4.24 ± 1.56	2.16 ± 1.38
Plasma protein concentration, g L^−1^			
Sherpas	67.5 ± 0.39	70.3 ± 0.32	68.1 ± 0.30
Lowlanders	67.8 ± 0.28	69.5 ± 0.49	68.9 ± 0.29
Total circulating protein mass, g			
Sherpas	194 ± 26	204 ± 30	163 ± 35
Lowlanders	195 ± 30	199 ± 35	149 ± 25
%HbCO, %			
Sherpas	2.02 ± 1.21	1.78 ± 0.57	2.67 ± 1.38
Lowlanders	1.97 ± 1.01	1.71 ± 1.00	3.06 ± 1.78

Absolute values measured during the baseline in Kathmandu and the two time points in the Everest Base Camp (EBC) are presented for all the participants tested at a given time point. Due to the large number of dropouts and resulting variation in participant numbers between time points, we did not perform statistical testing and present these absolute values for descriptive purposes only. EBC 1 and EBC 2 measurements were performed 2.3 ± 1.3 and 46.1 ± 4.3 days after arrival in the EBC, respectively, which was reached by foot ascent over the course of 5.0 ± 1.5 days. %HbCO, carboxyhemoglobin fraction in venous blood; ERC, Everest Base Camp; [Hb], venous hemoglobin concentration; BV, total blood volume; Hb_mass_, total hemoglobin mass; PV, plasma volume; RCV, total red cell volume; $\text{S}{\text{p}}_{{\text{O}}_{2}}$, peripheral oxygen saturation derived from pulse oximetry; erythropoietin, reticulocyte, and plasma protein concentrations as measured in venous blood.

### Hematocrit and Hemoglobin Concentration

**Figure 2.**
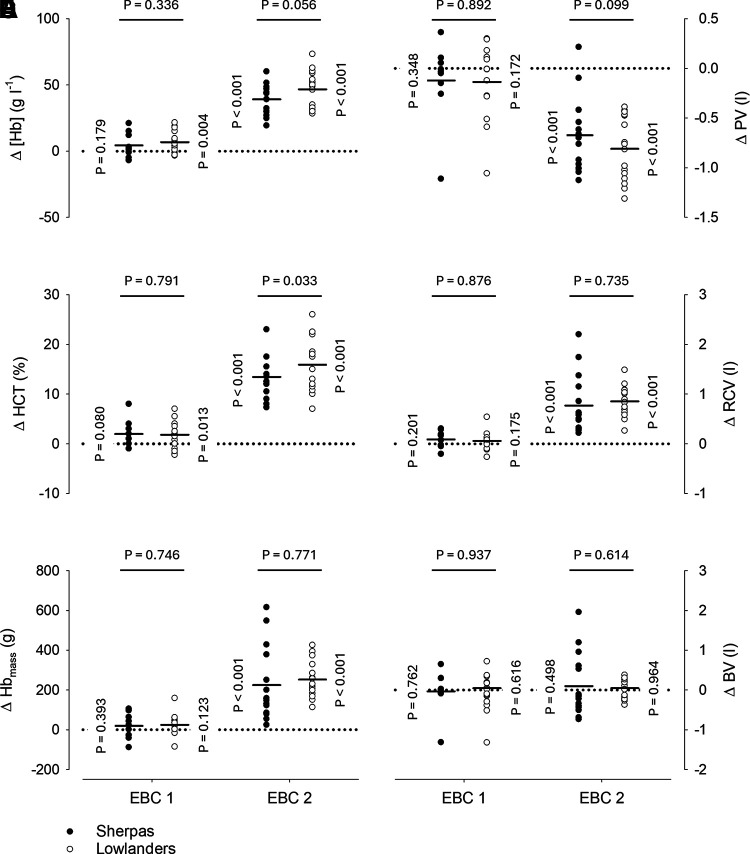
*A*–*F*: hematological changes in the Everest Base Camp (EBC). Circles represent individual changes from baseline during the two measurement time points in the EBC, whereas average changes are illustrated for Sherpas and lowlanders by horizontal lines. Vertical *P*-values correspond to the change from baseline within a given group and horizontal *P*-values to the comparison of changes between groups. A mixed model repeated measures (MMRM) analysis was used for statistical analysis with *P*-values corrected by Tukey’s method for multiple comparisons. EBC 1 (9 Sherpas and 14 lowlanders) and EBC 2 (15 Sherpas and 16 lowlanders) measurements were performed 2.3 ± 1.3 and 46.1 ± 4.3 days after arrival in the EBC, which was reached by foot ascent over the course of 5.0 ± 1.5 days. [Hb], venous hemoglobin concentration; BV, total blood volume; Hb_mass_, total hemoglobin mass; HCT, venous hematocrit; PV, plasma volume; RCV, total red cell volume; Δ, change from baseline.

Changes from baseline in [Hb] and hematocrit in the EBC are illustrated in Fig. 2, *A* and *B*, respectively. On EBC 1, [Hb] was not significantly changed in Sherpas (*P* = 0.179), whereas it was increased by 6.85 ± 8.46 g L^−1^ in lowlanders (*P* = 0.004, *d* = 0.6). On EBC 2, [Hb] was increased by 39.2 ± 11.7 (*d* = 3.2) and 46.7 ± 13.7 g L^−1^ (*d* = 3.7) in Sherpas and lowlanders, respectively (both *P* < 0.001). The [Hb] changes did not differ between groups on either EBC 1 (*P* = 0.336) or EBC 2 (*P* = 0.056). Hematocrit was also not significantly changed in Sherpas on EBC 1 (*P* = 0.080), while it was increased by 1.82 ± 3.00% in lowlanders (*P* = 0.013). On EBC 2, hematocrit was increased by 13.5 ± 4.2 and 15.9 ± 5.1% in Sherpas and lowlanders, respectively (both *P* < 0.001). The hematocrit changes were not different between groups on EBC 1 (*P* = 0.791) but were larger in lowlanders than in Sherpas on EBC 2 (*P* = 0.033).

### Hemoglobin Mass and Intravascular Volumes

Changes from baseline in Hb_mass_ and intravascular volumes in the EBC are presented in Fig. 2, *C*–*F*. While on EBC 1 Hb_mass_ changes were not significant in either Sherpas (*P* = 0.393) or lowlanders (*P* = 0.123), Hb_mass_ was increased on EBC 2 by 225 ± 183 (*d* = 1.5) and 252 ± 90 g (*d* = 1.6) in Sherpas and lowlanders, respectively (both *P* < 0.001). The Hb_mass_ changes did not differ between groups on either EBC 1 (*P* = 0.746) or EBC 2 (*P* = 0.771). Changes in PV on EBC 1 were also not significant in either Sherpas (*P* = 0.348) or lowlanders (*P* = 0.172), whereas on EBC 2 PV was reduced by 672 ± 377 (*d* = 1.6) and 810 ± 318 mL (*d* = 1.9) in Sherpas and lowlanders, respectively (both *P* < 0.001). The PV changes did not differ between the groups on either EBC 1 (*P* = 0.892) or EBC 2 (*P* = 0.099). Changes in total red cell volume paralleled those of Hb_mass_, with no significant changes observed on EBC 1 (Sherpas, *P* = 0.201; lowlanders, *P* = 0.175), and marked increases on EBC 2 (*P* < 0.001 for both groups) that were not different between groups (*P* = 0.735). No significant changes in total blood volume were observed in the EBC in either group (all *P* ≥ 0.498) and there were also no differences in the changes between groups (EBC 1, *P* = 0.937; EBC 2, *P* = 0.614).

### Erythropoietin and Reticulocytes

**Figure 3.**
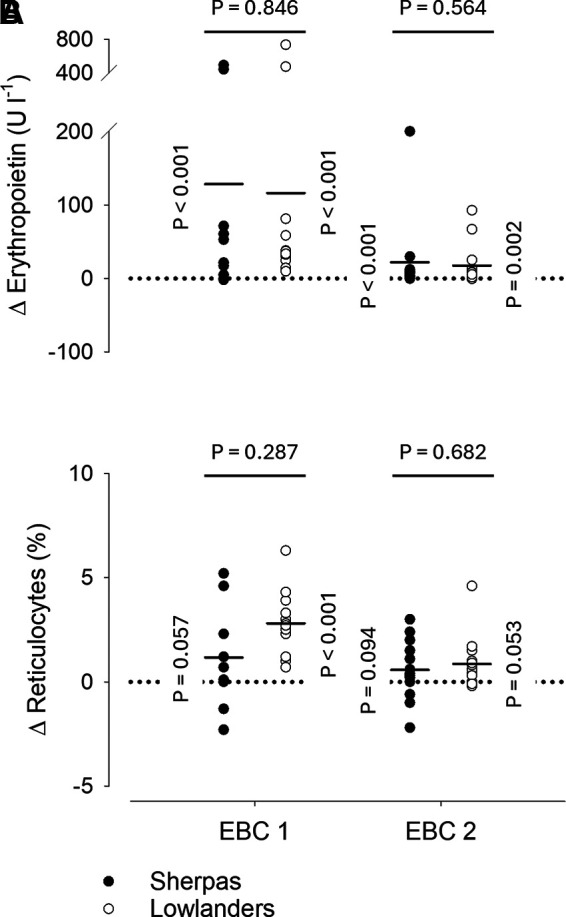
Changes in venous erythropoietin (*A*) and reticulocyte (*B*) concentrations in the Everest Base Camp (EBC). Circles represent individual changes from baseline during the two measurement time points in the EBC, whereas average changes are illustrated for Sherpas and lowlanders by horizontal lines. Vertical *P*-values correspond to the change from baseline within a given group and horizontal *P*-values to the comparison of changes between groups. A mixed model repeated measures (MMRM) analysis was used for statistical analysis with *P*-values corrected by Tukey’s method for multiple comparisons. EBC 1 (9 Sherpas and 14 lowlanders) and EBC 2 (15 Sherpas and 16 lowlanders) measurements were performed 2.3 ± 1.3 and 46.1 ± 4.3 days after arrival in the EBC, which was reached by foot ascent over the course of 5.0 ± 1.5 days. Δ, change from baseline; erythropoietin and reticulocyte concentrations as measured in venous blood.

Circulating erythropoietin concentration (Fig. 3*A*) was increased in both groups and on both time points in the EBC (all *P* ≤ 0.002) and this increase did not differ between groups on either EBC 1 (*P* = 0.846) or EBC 2 (*P* = 0.564). No significant change in circulating reticulocyte concentration (Fig. 3*B*) was detected on EBC 1 in Sherpas (*P* = 0.057), whereas there was an increase of 2.81 ± 1.57% in lowlanders (*P* < 0.001), that is, however, not different from the non-significant increase in Sherpas (*P* = 0.287). On EBC 2, there was no significant change in the reticulocyte concentration in either group (Sherpas, *P* = 0.094; lowlanders, *P* = 0.053) and no difference between the groups (*P* = 0.682).

### Plasma Proteins

**Figure 4.**
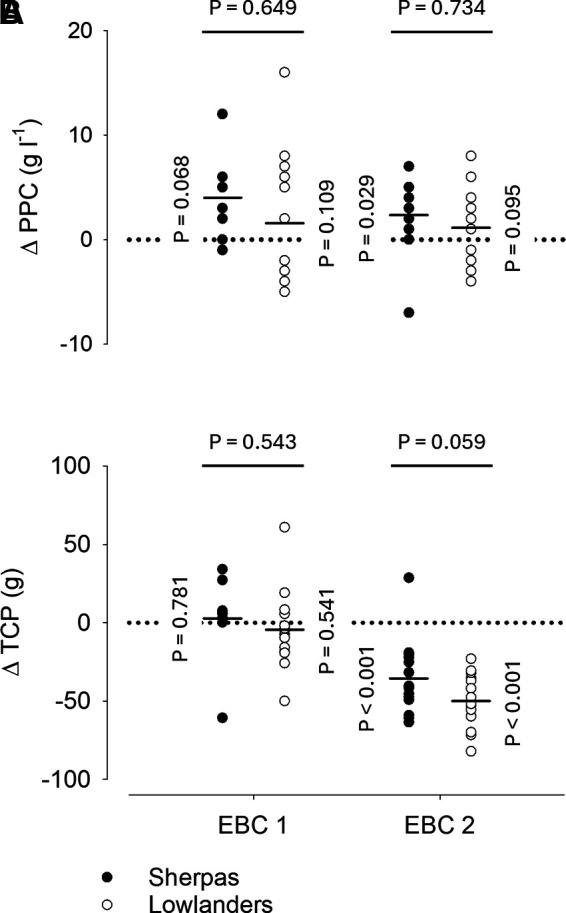
*A* and *B*: changes in plasma proteins in the Everest Base Camp (EBC). Circles represent individual changes from baseline during the two measurement time points in the EBC, whereas average changes are illustrated for Sherpas and lowlanders by horizontal lines. Vertical *P*-values correspond to the change from baseline within a given group and horizontal *P*-values to the comparison of changes between groups. A mixed model repeated measures (MMRM) analysis was used for statistical analysis with *P*-values corrected by Tukey’s method for multiple comparisons. EBC 1 (9 Sherpas and 14 lowlanders) and EBC 2 (15 Sherpas and 16 lowlanders) measurements were performed 2.3 ± 1.3 and 46.1 ± 4.3 days after arrival in the EBC, which was reached by foot ascent over the course of 5.0 ± 1.5 days. PPC, plasma protein concentration in venous blood; TCP, total circulating protein mass; Δ, change from baseline.

Changes from baseline in plasma protein concentration (Fig. 4*A*) on EBC 1 were not significant in either group (Sherpas, *P* = 0.068; lowlanders, *P* = 0.109) and not different between groups (*P* = 0.649). On EBC 2, plasma protein concentration was increased by 2.36 ± 0.33 g L^−1^ in Sherpas (*P* = 0.029), but not in lowlanders (*P* = 0.095). Nevertheless, also on EBC 2 no difference was detected between the changes in plasma protein concentration experienced by the two groups (*P* = 0.734). Changes in total circulating protein mass (the product of plasma protein concentration and PV, Fig. 4*B*) were not significant in either group (both *P* ≥ 0.541) and similar between groups on EBC 1 (*P* = 0.543). On EBC 2, total circulating protein mass was reduced by 35.5 ± 23.8 and 50.0 ± 16.9 g in Sherpas and lowlanders, respectively (both *P* < 0.001), and also this reduction did not differ between groups (*P* = 0.059).

## DISCUSSION

The major finding of this study is that Sherpas experience marked increases in Hb_mass_ and reductions in PV during extended exposure to 5,400 m altitude. These responses were similar to those observed in native lowlanders undergoing the same HA exposure, even though the resulting hemoconcentration tended to be somewhat smaller in the Sherpas. Moreover, the HA-induced increases in circulating erythropoietin were similar between the groups. These findings are in contrast to our hypotheses and do not support functionally relevant differences in the hematological response to HA exposure between Sherpas and lowlanders.

### Hemoglobin Concentration

Tibetan highlanders are considered to have a substantially lower [Hb] at HA than other populations (3). However, in the current study, acclimatization to 5,400 m altitude increased the [Hb] of Sherpas to 182 g L^−1^, which is comparable with the 190 g L^−1^ reached by the lowlander group, or to the 186 g L^−1^ previously measured in Danish lowlanders exposed for ∼10 wk to a similar altitude (14). It could be speculated that more extended HA exposure would have been required for the low [Hb] phenotype to develop in Sherpas but this is unlikely since the [Hb] of Sherpas on EBC 2 was identical to that measured in Tibetan nomads permanently living at the altitude of the EBC (15). Another explanation could be that pronounced [Hb] differences between Tibetans and acclimatized lowlanders occur only at more moderate altitudes, but this seems unlikely as well: a recent meta-analysis has reported [Hb] of Tibetans and Sherpas living between 3,000 and 4,000 m altitude to be 163 and 169 g L^−1^, respectively, whereas [Hb] of North Americans living at the same altitudes was 164 g L^−1^ (16). Of note, studies supporting a notably low [Hb] in Tibetans have often used South American highlanders (17, 18) or acclimatized Han Chinese (19, 20) as control populations. These two ethnicities seem to have a higher [Hb] at a given altitude than other populations (16), which could explain why the difference in the [Hb] of Tibetans was more pronounced than in the current study.

The aforementioned meta-analysis also predicts Tibetans and other Asian populations to increase [Hb] by ∼6.5 g L^−1^ with each 1,000 m of altitude gain (16), whereas in the current study, the [Hb] increase per 1,000 m corresponded to 9.8 and 11.8 g L^−1^ in Sherpas and lowlanders, respectively. The reason for this more pronounced increase in both groups is unclear. A chronically increased %HbCO due to smoking, air pollution, or indoor cooking can increase [Hb] (21), but this effect is unlikely to interact with the effect of HA (22). Moreover, the resting %HbCO changed little between the measurements in Kathmandu and in the EBC (Table 2). It seems more likely that the linear model used by the meta-analysis (16) does not apply to the very HA of the current study. In fact, others have used an exponential model for the [Hb] increase with altitude, thus predicting considerably larger increases at very HAs than the linear model (22).

### Hemoglobin Mass

The classic explanation for a low [Hb] in Tibetans is a blunted or absent erythropoietic response to HA exposure (23). In a recent, cross-sectional study, the Hb_mass_ of Sherpas at HA was indeed markedly lower than that of South American highlanders, but still higher than that of lowlanders at sea level (5). However, the number of participants in that study was small for a cross-sectional comparison and the observed differences in Hb_mass_ could have also, at least in part, reflected differences in lifestyle including physical activity (24). Another confounder could have been differences in the characteristics of the HA exposure between the populations, as the South Americans continuously resided at an altitude of 4,350 m, whereas many of the Sherpas worked as trekking guides and thus traveled between higher and lower altitudes on a regular basis. While the participant number in the present study was not larger, the assessment of HA-induced changes in Hb_mass_ in a longitudinal design constitutes a more sensitive analysis. Moreover, Sherpas and lowlanders underwent the same HA exposure where they lived, ate, and worked together, ruling out systematic differences in lifestyle, diet, and physical activity. We observed a 30% and 33% increase in Hb_mass_ on EBC 2 in Sherpas and lowlanders, respectively, which aligns with the predictions of a meta-analysis (25) and argues against a significantly blunted erythropoietic response to HA in Sherpas. It should be noted, however, that the average Hb_mass_ increase in Sherpas was driven upwards by two participants with an extremely large response (+615 and +548 g, respectively). One of these participants briefly lost the nose-clip during the CO rebreathing, which might have led to an overestimation of Hb_mass_, but in the participant with the largest Hb_mass_ increase no such incidence occurred. Interestingly, the two participants also exhibited markedly higher increases in erythropoietin than the other Sherpas on EBC 1 and, in case of the participant with the largest Hb_mass_ increase, also on EBC 2, providing a physiological explanation for their large Hb_mass_ increases. It should also be emphasized that even if these participants are removed from the analysis, the average increase in Hb_mass_ in Sherpas remains substantial (170 g). A blunted erythropoietic response of Sherpas to HA is further refuted by their increase in circulating erythropoietin on EBC 1, which was similar to that experienced by the lowlanders. The large interindividual variability in the erythropoietin response observed in both groups is in line with a previous study of lowlanders exposed to extreme altitude (26).

### Plasma Volume

In lowlanders acclimatized to HA, the increased [Hb] does not only reflect Hb_mass_ expansion but also a reduction in PV (1, 2). The PV of Sherpas at HA has recently been found to markedly exceed that of partially acclimatized lowlanders and Andean highlanders, and, at least numerically, that of lowlanders at low altitude (5), suggesting that HA exposure facilitates no reduction or even an increase in PV in Sherpas. This explanation is, however, not supported by the current results since the PV of Sherpas decreased in the EBC by almost 700 mL. It could be speculated that this PV contraction in Sherpas reflected chronic dehydration, rather than a response to hypoxia, but this is unlikely since beverages (i.e., tea and water) were readily available in the EBC. Moreover, the PV contraction was accompanied by a marked reduction in the total circulating protein mass, which constitutes the main mechanism underlying hypoxia-induced PV contraction (1, 27, 28). In the study reporting a large PV in Sherpas (5), measurements were performed in the Pyramid Research Laboratory (5,050 m), which was reached by most participants over the course of a multiday trek. Intravascular volumes were then assessed ∼7 days after the Sherpas’ arrival in the Pyramid (M. Stembridge, unpublished observations). This protocol resembles that of the EBC 1 measurement in the current study, where no PV contraction was observed in either group. Another study reported [Hb] 3–9 days after arrival in the Pyramid to be only 145 and 147 g L^−1^ in lowlanders and Sherpas, respectively (29), also indicating the absence of PV contraction in both groups. It is possible that the exercise associated with a multiday trekking ascent leads to a PV expansion that persists for several days and can overrule the opposing effect of HA exposure (30, 31). Whatever the explanation, it seems that an unchanged PV several days after foot ascent to >5,000 m is not a phenomenon that is exclusively found in Sherpas. Our results further demonstrate that Sherpas experience a normal PV contraction at HA if a longer exposure time is provided.

### Methodological Considerations

Due to the limited number of participants, we cannot exclude that the absence of significant differences between the hematological responses to HA of Sherpas and lowlanders reflects a Type II error. In fact, on EBC 2, the increase in [Hb] tended to be smaller, whereas the increase in hematocrit was significantly smaller in Sherpas than in lowlanders, suggesting a somewhat attenuated Hb_mass_ and/or PV response in Sherpas. The numerical differences between the groups in all the measured variables were, however, minor in comparison to the HA-induced changes and presumably of limited functional relevance. Another aspect to consider is that even the mild altitude of Kathmandu, where our participants lived before baseline measurements, can already evoke a slight increase in [Hb] (32). Nevertheless, this did clearly not prevent further hemoconcentration in response to exposure to higher altitudes as illustrated by the marked increases in [Hb] that both groups experienced in the EBC. It could also be speculated that the lowlanders in our study have, either through recurrent HA exposure or past genetic mixing, adopted a phenotype resembling that of Sherpas, thus preventing more substantial differences between the groups. We consider this unlikely, since, as detailed above, the increase in [Hb] in the lowlanders in the EBC was similar to that observed in Danish lowlanders at similar altitudes. Finally, we believe that the magnitudes of the Hb_mass_ and PV changes observed in the Sherpas in the EBC refute the paradigm of markedly blunted or absent erythropoietic and/or PV responses to HA in this population independently of the changes observed in the lowlanders.

A limitation of our study is that the exercise associated with the ascent to the EBC makes it difficult to isolate the effect of HA on the changes observed on EBC 1. We still consider the data collected at this time point relevant since, as detailed above, they provide an explanation for the large PV previously observed in Sherpas following a trekking ascent to a similar altitude (5). Moreover, it seems unlikely that exercise explained the marked increases in erythropoietin observed on EBC 1 since the effects of exercise on circulating erythropoietin are minimal (33). A further limitation is the inclusion of smokers, although, as discussed above, it seems unlikely that smoking interacts with the hematological response to HA (22), and since the number of smokers at baseline and on EBC 2 (where increases in Hb_mass_ were observed) was similar between groups. As reported in the methods, we also included some participants who transiently sojourned at lower altitudes between the EBC 1 and EBC 2 measurements. While the short descents of three participants to altitudes between 4,000 and 4,600 m seem unlikely to have affected our results, four participants undertook longer sojourns at lower altitudes, which could have attenuated their hematological changes on EBC 2. However, as three of these participants were Sherpas, such an attenuation should have, if anything, increased the difference between Sherpas and lowlanders on EBC 2. Finally, we were unable to include female participants since it is uncommon for women to work in the EBC. It is thus unclear whether our findings apply to female Sherpas.

### Perspective

A comparatively low [Hb] is considered a key adaptive trait of Tibetan highlanders that enhances their tolerance to live and exercise at HA (23). The most popular explanation for such a low [Hb] is that genetic adaptations in Tibetans suppress hypoxia sensing in the kidneys, thus preventing or at least attenuating renal erythropoietin release and the resulting Hb_mass_ expansion at HA (7, 34, 35). The broad acceptance of this explanation seems, however, premature since it lacks experimental confirmation: to our knowledge, no longitudinal study has ever confirmed HA-induced increases in [Hb] and/or Hb_mass_ to be smaller in Tibetans than in lowlanders exposed for the same duration to the same altitude. Studies comparing the erythropoietin response to hypoxia between Tibetans and lowlanders also provide limited support: in one study, the erythropoietin response to 8 h of hypoxia was smaller in a subgroup of Tibetans with the strongest genetic evidence for suppression of the oxygen sensing pathways than in the remaining Tibetans (35). Nevertheless, on average, the erythropoietin response of all the Tibetans was almost identical to that of the Han Chinese. Others observed circulating erythropoietin to be similar between Sherpas living at 3,440 m and native lowlanders at low altitudes (34). While this was interpreted as support for a blunted erythropoietin response to hypoxia in Sherpas, it should be considered that also in lowlanders erythropoietin returns to sea level values after only a few weeks of exposure to a similar altitude (36). The notion that a low [Hb] enhances the Tibetans’ capacity to exercise at HA is only supported by correlational evidence (4), and contrasts with studies reporting that experimentally reducing [Hb] does not improve or even decreases maximal or submaximal exercise capacity at HA in acclimatized lowlanders (37, 38). We hope that by challenging the established paradigms the current study will draw attention to this lack of direct evidence and inspire well controlled, mechanistic studies investigating the physiological consequences of the genetic adaptations that have been detected in Tibetans.

### Conclusions

Sherpas exposed for several weeks to very HA experience marked increases in Hb_mass_ and reductions in PV. These responses do not fundamentally differ from those experienced by native lowlanders, thus arguing against a distinct hematological response to HA in Tibetans. We propose that future studies comparing the hematological effects of HA between populations should preferably use a longitudinal design and match populations for lifestyle including diet and physical activity, as well as for the duration of exposure to the target altitude before testing.

## DATA AVAILABILITY

Data will be made available upon reasonable request.

## GRANTS

The travel costs associated with this study were partially covered by a “Short Mobility” grant (Autonomous Province of Bolzano—South Tyrol, Italy; Grant 170_2023). The Department of Innovation, Research, University and Museums of the Autonomous Province of Bozen/Bolzano covered Open Access publication costs.

## DISCLOSURES

No conflicts of interest, financial or otherwise, are declared by the authors.

## AUTHOR CONTRIBUTIONS

J.R., C.S., S.B., H.G., M.P.H., and S.T. conceived and designed research; J.R., C.S., S.B., S.B., S.S., A.S., and S.T. performed experiments; C.S. and P.R. analyzed data; J.R., C.S., S.B., H.G., P.R., M.P.H., and S.T. interpreted results of experiments; J.R. and C.S. prepared figures; C.S. drafted manuscript; J.R., C.S., S.B., S.B., S.S., H.G., P.R., M.P.H., A.S., and S.T. edited and revised manuscript; J.R., C.S., S.B., S.B., S.S., H.G., P.R., M.P.H., A.S., and S.T. approved final version of manuscript.
